# Long-term outcomes of elosulfase alfa enzyme replacement therapy in adults with MPS IVA: a sub-analysis of the Morquio A Registry Study (MARS)

**DOI:** 10.1186/s13023-025-04064-w

**Published:** 2025-10-30

**Authors:** Karolina M. Stepien, Barbara K. Burton, Michael B. Bober, Philippe M. Campeau, Carolyn Ellaway, Kaustuv Bhattacharya, Nathalie Guffon, David Hinds, Abigail Hunt, Alice Lail, Shuan-Pei Lin, Martin Magner, Elaine Murphy, Pascal Reisewitz, John J. Mitchell

**Affiliations:** 1 Northern Care Alliance NHS Foundation Trust, Salford, UK; 2 Ann & Robert H. Lurie Children’s Hospital of Chicago, Chicago, IL USA; 3 Nemours/Alfred I. DuPont Hospital for Children, Wilmington, DE USA; 4 CHU Sainte-Justine Research Center, Montreal, Quebec Canada; 5 Sydney Children’s Hospital Network, The Children’s Hospital at Westmead, Westmead, Sydney, NSW Australia; 6 Reference Centre of Inherited Metabolic Disease, Hospices Civils de Lyon, Lyon, France; 7 BioMarin Pharmaceutical Inc., Novato, CA USA; 8 MacKay Children’s Hospital, Taipei, Taiwan; 9 Department of Pediatrics and Inherited Metabolic Disorders, General University Hospital and First Faculty of Medicine, Charles University, Prague, Czech Republic; 10 National Hospital for Neurology and Neurosurgery, London, UK; 11 McGill University Health Centre, Montreal, Quebec Canada

**Keywords:** Elosulfase alfa, Mucopolysaccharidosis IVA, Clinical outcomes, Patient-reported outcomes, Real-world evidence

## Abstract

**Background:**

Mucopolysaccharidosis (MPS) IVA is a rare disease with substantial, multisystemic morbidity. We assessed real-world safety and effectiveness of the enzyme replacement therapy (ERT) elosulfase alfa in patients with MPS IVA in the multinational, observational Morquio A Registry Study (MARS) who initiated ERT in adulthood (aged ≥ 18 years).

**Methods:**

Patients were enrolled between September 2014 and February 2022; urinary keratan sulfate (uKS), 6-minute walk test (6MWT) distance, forced expiratory volume in 1 s (FEV_1_), forced vital capacity (FVC), EuroQoL-5D-5L (EQ-5D-5L) score, and safety were assessed during routine care.

**Results:**

As of February 13, 2022, 90 patients who initiated ERT had enrolled (median exposure: 5.6 years; median age at first ERT: 27.8 years). Reductions from baseline in uKS levels were sustained over mean follow-up of 5.4 years (mean percent change: -52.9%; *p* < 0.0001). In patients with available data, mean change in 6MWT distance was + 15.8 m (*p* = 0.3627) over a mean follow-up of 5.8 years. FEV_1_ and FVC remained stable over mean follow-up of 5.3 years (mean change: 0.0 L for both). The mean change from baseline in EQ-5D-5L index score was + 0.1 after 1 year of treatment. Thirty-four patients (39.5%) had ≥ 1 adverse event (AE), 23 patients (26.7%) had ≥ 1 serious AE, and 10 (11.6%) had ≥ 1 drug-related AE (infusion-related reactions [*n* = 3; 3.5%], pyrexia [*n* = 2; 2.3%]). Eight deaths occurred; none were deemed treatment related.

**Conclusions:**

Real-world data collected from MARS suggest that patients with MPS IVA who initiated ERT in adulthood remained stable over 7 years of follow-up. No new safety signals were identified.

**Supplementary Information:**

The online version contains supplementary material available at 10.1186/s13023-025-04064-w.

## Introduction

Mucopolysaccharidosis (MPS) IVA, also known as Morquio A syndrome (OMIM #253000), is a rare, inherited lysosomal storage disorder in which deficient activity of the enzyme *N*-acetylgalactosamine-6-sulfatase (GALNS; EC: 3.1.6.4) causes accumulation of the glycosaminoglycans keratan sulfate and chondroitin-6-sulfate in multiple tissues and organs [1–4]. This results in progressive development of the multisystemic signs and symptoms of MPS IVA, including skeletal dysplasia, impaired respiratory function, cardiac valve abnormalities, impaired vision and hearing, neurologic complications, and hepatosplenomegaly [1–5].

MPS IVA is a heterogeneous disease, with a spectrum of clinical presentations generally grouped into two main phenotypes [1–3, 5]. Patients with a “classical” phenotype have a severe, rapidly progressive disease, early onset typically diagnosed during childhood, with mortality expected by the second or third decade of life if left untreated [1–5]. In contrast, patients with a “non-classical” phenotype often have attenuated progression, later disease onset, and diagnostic delays due to atypical symptom presentation, with extended survival into the seventh decade of life [1, 3, 5, 6]. Current guidelines recommend the initiation of long-term elosulfase alfa (Vimizim^®^, BioMarin Pharmaceutical Inc., Novato, CA), a recombinant human GALNS enzyme replacement therapy (ERT) approved for the treatment of MPS IVA [7, 8], in all patients as soon as possible after a confirmed diagnosis [9].

The efficacy and safety of elosulfase alfa was evaluated in the pivotal 24-week, placebo-controlled, Phase 3 MOR-004 study (NCT01275066) in pediatric and adult patients with MPS IVA [10], and in the 96-week open-label extension MOR-005 study (NCT01415427) [11, 12]. After 24 weeks of treatment, elosulfase alfa (2 mg/kg every week) was associated with significant improvements in endurance (as measured by the 6-minute walk test [6MWT]), reductions in urinary keratan sulfate (uKS) levels, and numeric improvements in respiratory function [10] and ability to perform activities of daily living (ADLs) compared with placebo [13], which were sustained for up to 120 weeks of treatment [11, 12, 14, 15]. In contrast, comparable untreated patients from the Morquio A Clinical Assessment Program (MorCAP) natural history (NH) study (NCT00787995) showed reductions in endurance and no improvements in uKS over a similar period of time, with patients >14 years of age having worsening of respiratory function [5]. Elosulfase alfa was generally well tolerated in these clinical trials [10, 12, 14]. Early intervention with elosulfase alfa is likely to change the course of the disease in patients with MPS IVA [9]. Sibling case studies have reported that early recognition, diagnosis, and initiation of treatment may slow irreversible disease progression, reduce symptom severity, and improve patient-reported outcomes (PROs) [16–18].

While the clinical benefits of early ERT for patients with MPS IVA are well established, the benefits of treatment initiation in patients already in adulthood are less well defined. The clinical trials of elosulfase alfa predominantly enrolled pediatric patients (median baseline age in MOR-004: 11.9 years) [10]. However, a post hoc analysis of the MOR-005 study in patients who initiated treatment during adulthood (*n* = 37) demonstrated increased endurance, stability of respiratory function, and improvements in performance of ADLs [14].

The observational Morquio A Registry Study (MARS), initiated in 2014, was designed to collect long-term data on patients with MPS IVA for up to 10 years, regardless of ERT treatment status [19]. Data collected in MARS provide a means to assess the long-term safety and effectiveness of elosulfase alfa in a real-world setting, outside of clinical trials with limited follow-up and restrictive inclusion and exclusion criteria. Data from the first 6 years of MARS have provided valuable real-world evidence for long-term stabilization of endurance and respiratory function among patients treated with elosulfase alfa [19]. However, understanding the long-term benefits of initiating ERT in adults with pre-existing disease burden remains an important clinical question as prior studies, including the initial MARS study, were primarily conducted in pediatric patients [19].

The aim of this sub-analysis was to assess the real-world safety and effectiveness of elosulfase alfa, and PROs, in patients with MPS IVA with up to 7 years of observational data in MARS who initiated treatment with elosulfase alfa in adulthood.

## Materials and methods

### Study design and patients

This observational analysis used data collected in MARS, a multicenter, multinational study of patients with MPS IVA. The overall MARS study design has been previously reported [19]. The study protocol was reviewed and approved by all applicable institutional review boards and ethics committees for participating study sites. Written informed consent was obtained from all patients before study enrollment. Baseline and follow-up data were collected from clinical assessments, PROs, and safety assessments that were performed as part of routine care.

### Analysis population and definitions

Patients with a diagnosis of MPS IVA confirmed by evidence of reduced GALNS enzyme activity or genetic testing were eligible for enrollment in MARS. This analysis included patients enrolled over the first 7 years of MARS, between September 2014 and February 2022, who initiated treatment with elosulfase alfa at ≥ 18 years of age. Registry Entry was defined as the date of signed consent. Assessments performed at Registry Entry were defined as those performed at the closest data point within the time window from 12 months before Registry Entry to 90 days after. Index was defined as the date of first exposure to elosulfase alfa, which may have occurred before or after Registry Entry. Baseline assessment was defined as the data point closest to Index, within the time frame from 12 months before Index to 30 days after. For measurements of uKS, baseline assessment was restricted to data points occurring at Index or within the 12 months prior.

Safety outcomes were described among all patients who received at least one dose of elosulfase alfa during MARS. Study investigators were directed to report: all serious adverse events (SAEs); all adverse events (AEs) occurring within 24 h of elosulfase alfa infusion; all non-serious adverse reactions to elosulfase alfa; any pregnancy; any unusual failure in efficacy; and all occurrences of spinal cord compression.

### Outcomes and statistical analyses

Demographics and clinical characteristics of patients at Registry Entry were summarized using descriptive statistics, including means, standard deviations, medians, ranges or interquartile ranges (IQRs), or counts and percentages, as dictated by data type. Change and/or percentage change from pre-ERT baseline in clinical outcome variables—normalized uKS, 6MWT distance, forced expiratory volume in 1 s (FEV_1_), and forced vital capacity (FVC)—were summarized for patients with both a baseline and follow-up measurement recorded at the relevant time point.

Paired *t*-tests for change or percentage change in clinical outcome variables from pre-ERT baseline to last on-treatment measurement were two-sided at the 0.05 alpha level. For 6MWT measurements, where it was recorded that the patient was physically unable to perform the test, a distance of 0 m was imputed, consistent with previous clinical and real-world analyses [10, 12, 19]. No imputation was performed for the values of other outcomes assessed.

Quality of life (QoL) was assessed using the EuroQoL-5D-5L (EQ-5D-5L), a generic QoL instrument that has been recommended for assessing disease burden in patients with MPS IVA [9, 20]. The scale comprises five domains: mobility, self-care, usual activities, pain/discomfort, and anxiety/depression. Each domain contains a rating scale composed of five levels: no problems, slight problems, moderate problems, severe problems, and unable to/extreme problems. EQ-5D-5L health states can be represented by a single summary index “utility” value, ranging from 1 (representing full health) to 0 (representing death) [20, 21]. For this analysis, the summary index was computed using the EQ-5D value set for the UK [21]. Change from baseline in EQ-5D-5L index score was assessed for all patients with a baseline measurement and a follow-up measurement recorded at the relevant time point. Descriptive results are presented; no formal statistical analyses were performed. If a patient had missing data for 6MWT because they were physically unable to perform the test, a value of 0 m was imputed. Data for all other outcomes are shown as observed.

AEs were assessed for all patients who received at least one dose of elosulfase alfa during MARS and include all study drug-related AEs and SAEs occurring after Index or Registry Entry (whichever occurred later). AEs were coded using version 23.1 of the Medical Dictionary for Regulatory Activities and are summarized as numbers and percentages of patients having events.

## Results

### Patients

As of February 13, 2022, a total of 90 patients who initiated elosulfase alfa at ≥ 18 years of age had enrolled in MARS. Patient demographics and clinical characteristics at Registry Entry are summarized in Table 1. The median standing height was 108.5 cm (range: 88.0–158.2 cm); although phenotype was not directly assessed during MARS, the range of baseline height data indicates that the population was heterogeneous.

**Table 1:** Patient demographics and clinical characteristics at Registry Entry

	ERT initiated ≥ 18 years (*n* = 90)
Sex, *n* (%)	
Female	56 (62.2)
Male	34 (37.8)
Race, *n* (%)	
White	61 (67.8)
Asian	14 (15.6)
Black or African American	2 (2.2)
Other	6 (6.7)
Missing/Not reported	7 (7.8)
Age at diagnosis, years	
Mean (SD)	13.1 (14.98)
Median (range)	4.8 (0.6–63.4)
Characteristics at Registry Entry	
Age, years	
Mean (SD)	34.4 (12.37)
Median (range)	31.3 (19.0–73.5)
Standing height, cm	
Overall	*n *= 58
Mean (SD)	115.2 (20.74)
Median (range)	108.5 (88.0–158.2)
Female	*n *= 43
Mean (SD)	111.8 (19.27)
Median (range)	101.0 (89.0–154.0)
Male	*n *= 15
Mean (SD)	125.2 (22.21)
Median (range)	128.0 (88.0–158.2)
Weight, kg	*n *= 79
Mean (SD)	35.7 (14.00)
Median (range)	34.0 (19.0–83.9)
Normalized uKS, µg/mg	*n* = 49
Mean (SD)	5.8 (5.35)
Median (range)	4.1 (1.4–32.8)
6MWT, m	*n *= 68
Mean (SD)	161.3 (164.39)
Median (range)	121.5 (0.0–543.0)

Abbreviations: 6MWT = 6-minute walk test, ERT = enzyme replacement therapy, SD = standard deviation, uKS = urinary keratan sulfate

Where data were not available for all patients for a given characteristic, the number with data (*n*) has been reported separately

The total median ERT exposure was 5.6 years (range: 3.2–8.1 years), with a median exposure during MARS of 4.3 years (range: 0.0–6.9 years) (Table 2). The median age at ERT initiation was 27.8 years (range: 18.9–68.7 years). Most patients (*n* = 67; 74%) had initiated treatment before Registry Entry. Over the follow-up period included in this analysis, 24 patients were considered to have discontinued ERT (i.e. had a recorded reason for discontinuation and no record of restart of treatment before the analysis cut-off date). Reasons for discontinuation were: patient death (*n* = 7), patient choice (*n* = 7), coronavirus disease 2019 (COVID-19; *n* = 4), financial/logistical reasons (*n* = 4), and AEs (*n* = 2), respectively.

**Table 2:** ERT exposure

	ERT initiated ≥ 18 years (*n* = 90)
Age at ERT initiation, years	
Mean (SD)	32.6 (12.11)
Median (IQR)	27.8 (22.6–38.6)
Min, max	18.9, 68.7
Total ERT exposure*, years	
Mean (SD)	5.4 (3.00)
Median (IQR)	5.6 (3.2–8.1)
Min, max	0.0, 10.4
Total ERT exposure in MARS†, years	*n* = 86
Mean (SD)	3.9 (1.98)
Median (IQR)	4.3 (2.6–5.6)
Min, max	0.0, 6.9

Abbreviations: ERT = enzyme replacement therapy, IQR = interquartile range, MARS = Morquio A Registry Study, Max = maximum, Min = minimum, SD = standard deviation

Where data were not available for all patients for a given characteristic, the number with data (*n*) has been reported separately

*Total exposure (before or after Registry Entry) is calculated as the date of last ERT treatment minus the date of first ERT treatment

†Exposure during MARS is calculated as the date of last ERT treatment minus the date of first ERT treatment or Registry Entry, whichever is later

### Effectiveness

#### Clinical outcomes

**Fig. 1:**
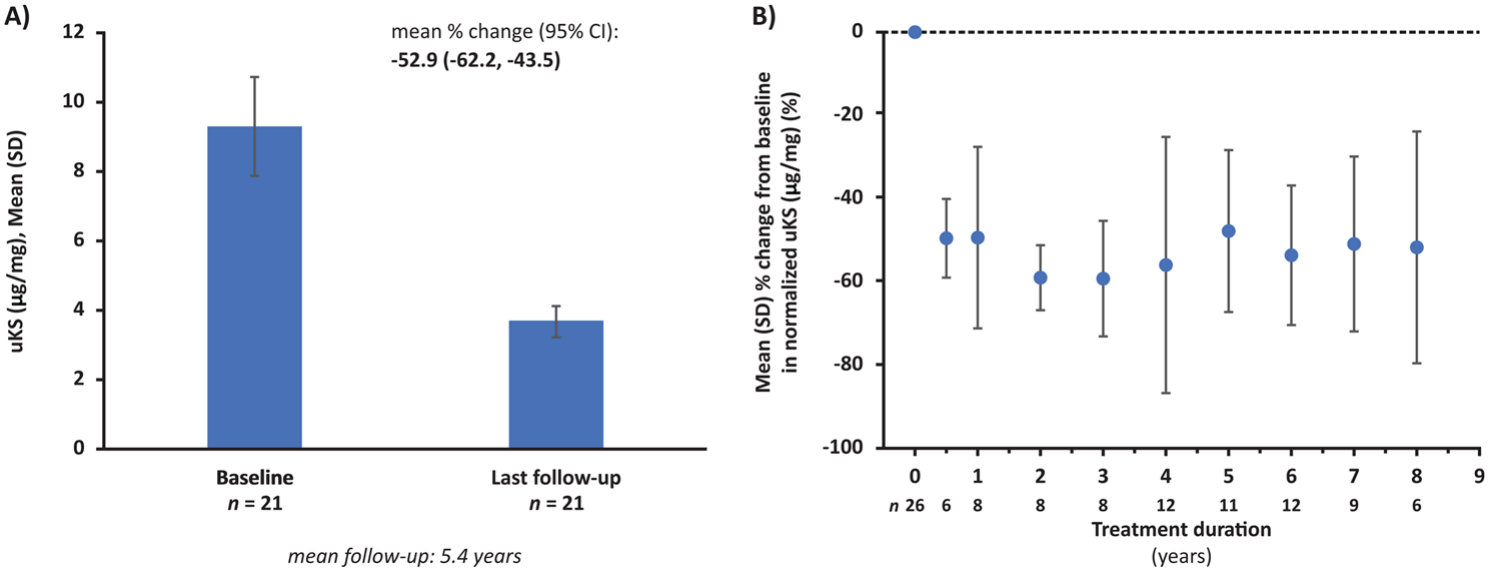
Change in normalized uKS from baseline to last follow-up (**A**) and by treatment duration (**B**). Abbreviations: CI = confidence interval, SD = standard deviation, uKS = urinary keratan sulfate

Within 6 months, normalized uKS levels declined rapidly from pre-ERT baseline, and the decrease was sustained thereafter. Mean percentage change from baseline to last follow-up in uKS level was -52.9% (95% confidence interval [CI]: -62.2, -43.5; *p* < 0.0001), over a mean follow-up of 5.4 years (Fig. 1).

**Fig. 2:**
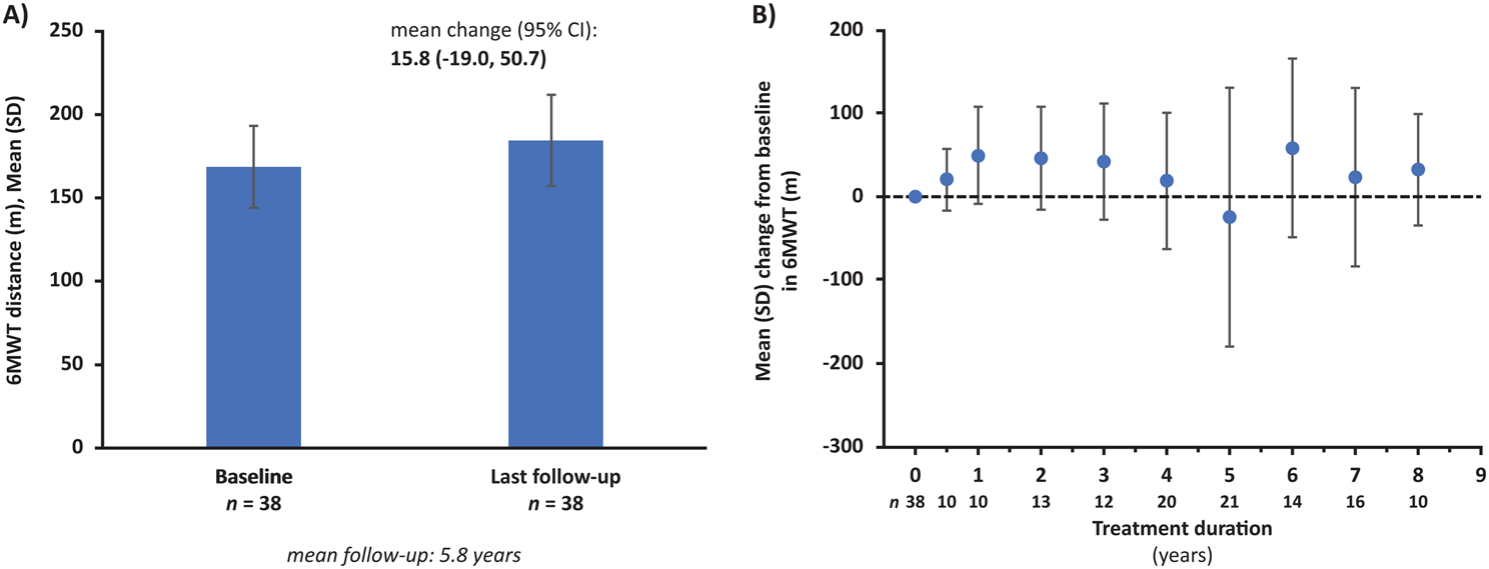
Change in 6MWT distance from baseline to last follow-up (**A**) and by treatment duration (**B**). Abbreviations: 6MWT = 6-minute walk test, CI = confidence interval, SD = standard deviation

Over a mean follow-up of 5.8 years, the mean change in 6MWT distance from a pre-ERT baseline of 168.7 m to the last follow-up was + 15.8 m (95% CI: -19.0, 50.7; *p* = 0.3627) (Fig. 2A). 6MWT remained generally stable throughout follow-up (Fig. 2B).

**Fig. 3:**
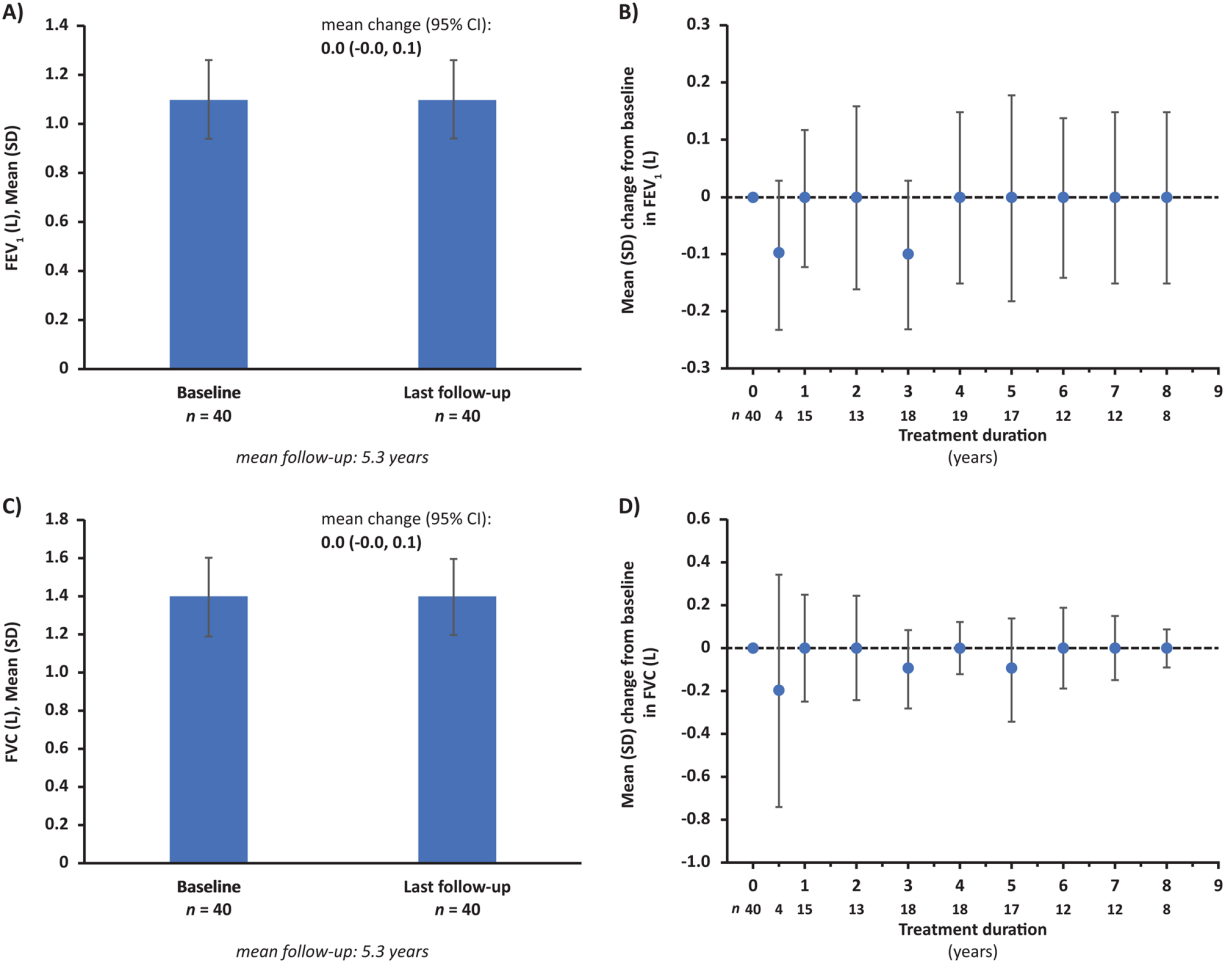
Change in respiratory function from baseline to last follow-up (**A**, **C**) and by treatment duration (**B**, **D**). Abbreviations: CI = confidence interval, FEV_1_ = forced expiratory volume in 1 s, FVC = forced vital capacity, SD = standard deviation

Respiratory parameters remained stable throughout follow-up, with a mean change from pre-ERT baseline (mean FEV_1_: 1.1 L; mean FVC: 1.4 L) to last follow-up of 0.0 L (95% CI: -0.0, 0.1) for both FEV_1_ and FVC, respectively, over a mean follow-up of 5.3 years (Fig. 3).

#### Patient-reported outcomes

**Fig. 4:**
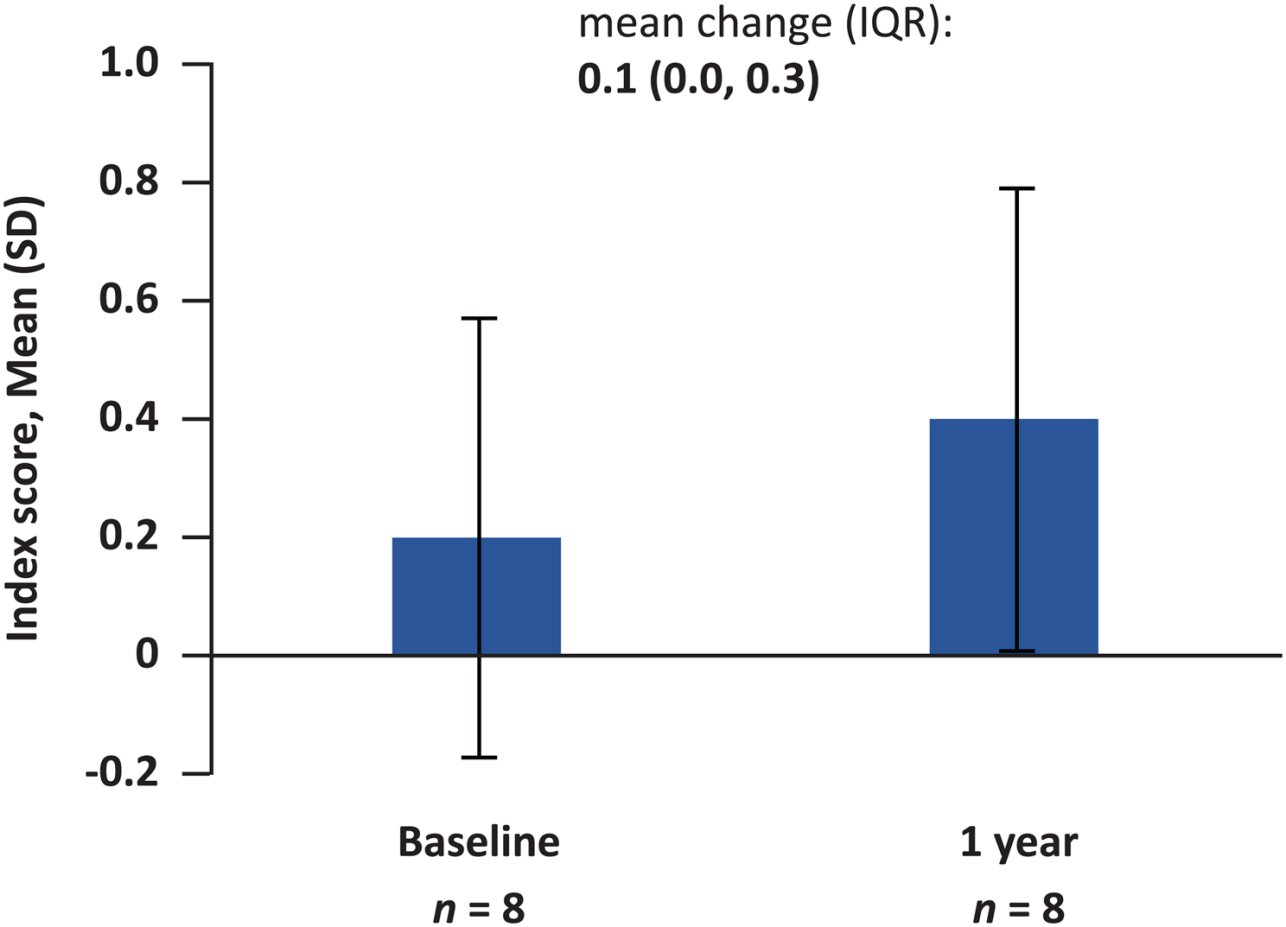
Change in EQ-5D-5L index score from baseline to 1 year. Abbreviation: EQ-5D-5L = EuroQoL-5D-5L

Pre-ERT baseline EQ-5D-5L index scores were available for 14 patients. The mean index score at baseline was 0.4 (IQR: 0.1–0.8), indicating general impairment. Among patients with available baseline and follow-up data, a mean increase from baseline of + 0.1 (IQR: 0.0–0.3) was observed after 1 year of treatment (*n* = 8) (Fig. 4).

Change from pre-ERT baseline in EQ-5D-5L domain scores indicated potential improvements in ADLs and pain, but limited or no improvement in mobility and ability to self-care (Supplemental Fig. S1). Anxiety was rarely reported by patients as a problem at baseline, and little change was observed in this domain over follow-up (Supplemental Fig. S1).

### Safety

The incidence of AEs, SAEs, and drug-related AEs are summarized in Table 3. Thirty-four patients (39.5%) had at least one AE; 19 AEs in 10 patients (11.6%) were deemed to be related to elosulfase alfa. Most patients who had study drug-related AEs had mild or moderate AEs (Grade 1 or 2: *n* = 9; 90.0%), and the most common study drug-related AEs were infusion-related reactions (*n* = 3; 3.5%) and pyrexia (*n* = 2; 2.3%). Overall, 23 patients (26.7%) had SAEs, with the majority of these patients having Grade 3 SAEs (*n* = 10; 43.5%). Two Grade 3 study drug-related gastrointestinal SAEs were reported in the same patient: one case of gastritis and one case of abdominal pain. Two AEs led to permanent study drug discontinuation: one event of infusion-related reaction (Grade 2) and one event of administration-site discomfort (Grade 1). Incident/worsening of spinal cord compression (one Grade 1 event, eight Grade 3 events) occurred in 9 patients (10.5%), and incident/worsening of cervical cord compression (three Grade 1 events, five Grade 2 events, four Grade 3 events) occurred in 11 patients (12.8%). All compression events were unrelated to treatment.

**Table 3:** Summary of AEs

Incidence, *n* (%)	ERT-treated (*n* = 86)
≥ 1 AE	34 (39.5)
≥ 1 study drug-related AE	10 (11.6)
Infusion-related reaction	3 (3.5)
Pyrexia	2 (2.3)
Upper abdominal pain	1 (1.2)
Diarrhea	1 (1.2)
Gastritis	1 (1.2)
Nausea	1 (1.2)
Discomfort	1 (1.2)
Hot flush	1 (1.2)
Hypertension	1 (1.2)
Hypersensitivity	1 (1.2)
Dyspnea	1 (1.2)
Urticaria	1 (1.2)
Abdominal pain	1 (1.2)
≥ 1 SAE	23 (26.7)
≥ 1 study drug-related SAE	1 (1.2)
Gastritis	1 (1.2)
Abdominal pain	1 (1.2)
AE leading to permanent study drug discontinuation	2 (2.3)
Deaths	8 (9.3)

Abbreviations: AE = adverse event, ERT = enzyme replacement therapy, SAE = serious adverse event

Eight patients (9.3%) died during MARS; the most frequently reported cause of death was respiratory failure. In these patients, the mean age at time of death, mean age at ERT initiation, and mean length of time on treatment was 35.3 years, 31.4 years, and 4.0 years, respectively. No deaths were assessed as related to elosulfase alfa (Table 4).

**Table 4:** Prior respiratory function, cause of death, and age at death during MARS

Cause of death	Sex	Age at ERT initiation,years	Age at death, years	Last recorded respiratory function	
				FEV_1_, L	FVC, L
Respiratory failure	F	37.2	38.7	0.82	0.62
Respiratory insufficiency	F	24.0	27.8	0.37	0.33
Respiratory failure	M	18.9	27.2	0.92	0.40
Cardiac arrest	M	22.3	25.6	0.55	0.46
Cardiac arrest	M	24.3	26.5	0.66	0.47
Respiratory failure	F	62.0	65.2	0.39	0.39
Respiratory failure	F	27.2	31.8	ND	ND
COVID-19	F	34.9	39.9	ND	ND

Abbreviations: COVID-19 = coronavirus disease 2019, ERT = enzyme replacement therapy, F = female, FEV_1_ = forced expiratory volume in 1 s, FVC = forced vital capacity, M = male, MARS = Morquio A Registry Study, ND = not determined

Cause of death was assessed by the investigator and an autopsy may not have been performed/reported

## Discussion

Understanding the benefits of initiating long-term ERT in adults with pre-existing disease burden is an important clinical question since patients with MPS IVA generally initiate treatment in childhood. The results from this sub-analysis of data from MARS showed that patients’ endurance and respiratory function appeared stable over the follow-up period, while sustained reductions in uKS were observed. Furthermore, safety data were generally consistent with the overall MARS analysis [19], with no new safety signals identified.

uKS is a key biomarker for MPS IVA, and previous studies have shown that higher levels of uKS correlate with more severe clinical impairment [3, 22]. The results of this sub-analysis show that initiation of elosulfase alfa in adulthood results in rapid reductions in uKS that are sustained over time. Similar sustained reductions were observed in the overall MARS analysis [19], therefore demonstrating the long-term, continued action of elosulfase alfa in patients with MPS IVA.

A mean change of +15.8m in 6MWT distance from baseline was observed over a mean follow-up of 5.8 years; however, this change was not statistically significant. While not conclusive, this suggests that ERT stabilized endurance in adults compared with the natural history of MPS IVA, in which a consistent decline in 6MWT has been observed [5]. Similar stabilization of endurance was observed with elosulfase alfa in the overall MARS analysis (mean change: -6 m) over 5.5 years [19]. The slight differences observed in the adult cohort versus the overall MARS population are likely due to the possible phenotypic variability of these populations, whereby patients who initiate ERT during adulthood may have a more attenuated and slowly progressive non-classical phenotype, and therefore perform better on the 6MWT. A post hoc analysis of data from adult patients in MOR-005 also demonstrated improvement in 6MWT of similar magnitude to the present analysis, with an increase of + 30.5 m observed after 2 years of treatment. The same post hoc analysis used data from matched untreated adults in the MorCAP NH study, showing stability of 6MWT (mean change: +6.5 m [well within the standard error of 15.9 m]) over a similar time period [14]. The long-term benefits of ERT on endurance were also shown in a cross-sectional analysis using data collected in the MARS and MorCAP studies, in which participants were matched by age at the time of 6MWT assessment in order to compare ERT-treated and untreated participants. This analysis showed a consistent positive association between ERT exposure and 6MWT in multivariate regression analyses adjusting for confounding factors [23].

Our cohort exhibited pre-existing impairment in respiratory function at pre-ERT baseline, which stabilized on ERT through to last follow-up, similar to observations from a previous analysis of the MARS adult population [19]. These findings concur with those from a post hoc analysis of MOR-005, demonstrating stability of respiratory function in patients initiating treatment at ≥ 18 years of age [14, 19], and indicate a treatment benefit of ERT in preventing further worsening of respiratory function, which is known to gradually worsen without treatment in MPS IVA [5]. In contrast, patients who initiate treatment with elosulfase alfa before 18 years of age generally demonstrate improved respiratory function [19]. This contrast is likely partly resultant from growth-related increases in respiratory parameters through childhood and adolescence not expected during adulthood, as well as the impact of irreversible, established physiologic manifestations of MPS IVA in adult patients. However, results from subgroup analyses performed on the MOR-005 cohort show that analyzing respiratory outcomes in patients treated above a lower age cut-off (14 years) demonstrated improvements with elosulfase alfa over 2 years of treatment, compared with a decline in untreated patients in the MorCAP study [11]. Furthermore, in some adult patients with a slowly progressive non-classical phenotype, stability of respiratory parameters could partly reflect maintenance of normal respiratory function [2, 6]. Further research is required to determine the age threshold for improvements in respiratory outcomes with ERT and to explore the possibility of treatment preserving respiratory function in patients with a slowly progressive non-classical phenotype.

MPS IVA phenotype has often been described using height as a proxy marker for classical versus non-classical disease; although phenotype was not a captured characteristic in the MARS cohort, comparisons of baseline heights of patients in the cohort with published growth curves for patients with MPS IVA can provide an estimation of the proportion of patients with classical versus non-classical phenotypes (defining classical phenotype as height ≤ 50th percentile) [24, 25]. On this basis, approximately 50% of female patients and a slightly lower proportion of male patients in the MARS cohort had a final adult height consistent with the classical phenotype of MPS IVA. Irrespective of phenotype, our treated cohort had long-term stabilization of endurance and respiratory function, outcomes known to otherwise progressively deteriorate [5], highlighting the importance of rapid treatment initiation even in patients for whom MPS IVA has presented and/or been diagnosed later in life. Longer follow-up will be required to confirm the durability of these findings.

Among patients with available baseline and follow-up data, there were numeric differences in EQ-5D-5L index scores, primarily in two domains that were most impaired at baseline: the pain and activities domains. There was negligible impact of treatment on the anxiety domain, although most patients indicated no or only slight problems with anxiety at baseline, so substantial improvements in this domain were unlikely. Minimal/no improvements were also observed in the self-care domain, although most patients also reported no or only slight problems with self-care at baseline (with the exception of 2 patients who were severely affected and remained so throughout follow-up). Limited/no improvements were observed in the mobility domain, which was expected given that this domain was likely to be one of the most affected by pre-existing physical disease manifestations in this adult population [26], demonstrated by the greater impairment of this domain at baseline.

Longer follow-up of more patients in MARS could allow for development of a more robust data set, allowing for stronger conclusions to be drawn regarding any clinically relevant benefits of initiating treatment with elosulfase alfa in adulthood.

Safety was largely consistent with the previous overall MARS analysis, with a slightly lower incidence of drug-related AEs and SAEs (11.6% and 1.2%, respectively) compared with the overall MARS population (12.4% and 1.6%, respectively), and no new or unexpected safety signals in this adult population. Eight patients who initiated ERT as adults died during MARS, with the most common cause of death being respiratory failure—a leading primary cause of death in patients with MPS IVA [27] and unrelated to treatment. Of the patients whose cause of death was attributed to respiratory failure, 2 patients had a history of using continuous/bilevel positive airway pressure. Other causes of patient mortality reported in this analysis include cardiac arrest, respiratory insufficiency, and COVID-19 infection, all of which likely reflect the increased risk of mortality from established, irreversible multisystemic manifestations of MPS IVA.

One limitation of this study was that data could not be compared to a control group, preventing statistical analyses of comparative effectiveness. The MARS study was designed to provide insights reflective of real-world MPS IVA treatment [19]. Because MARS was initiated shortly after the approval of elosulfase alfa for MPS IVA in 2014 [7, 8], the majority of patients enrolled in MARS received ERT already, or started ERT shortly after entry [19]. Consequently, the size of the ERT-naïve adult cohort was too small to be used as a control group for this analysis. Additionally, of the ERT-naïve population that has been identified from the overall MARS cohort, notable differences in disease characteristics have been reported compared with ERT-treated patients. For example, at the time they entered MARS, more than 50% of ERT-naïve patients were unable to complete the 6MWT, as opposed to < 10% of ERT-treated patients. These findings suggest that untreated patients enrolled in MARS might reflect either end of the MPS IVA phenotypic spectrum, ultimately restricting comparisons between our treated cohort and ERT-naïve adult patients in MARS [19]. Given that ERT has been shown to delay the progressive worsening of endurance in MPS IVA [10, 12], it is also probable that MARS participants aged ≥ 18 years who are still able to walk are more likely to have received ERT from an earlier age. Regardless of relative benefit on physical endurance, we observed a positive impact on other outcomes of MPS IVA in our analysis, highlighting the wider importance of initiating treatment with ERT even in adulthood.

No subgroup analyses based on phenotype severity (e.g. stratification of patients by age at diagnosis or height) were performed due to the relatively small sample size in this analysis. Another limitation was that minimal data were available at baseline for mobility and surgical measures, including oxygen dependence, use of mobility aids, and surgical history, as a result of the real-world nature of the study. Available surgical data in these adult patients are likely incomplete as these rely on patient recall and/or medical records, which can be limited if the patient has changed provider.

As data were not collected following a standardized schedule as they would be in a clinical trial, results for any given time point were restricted to patients with both baseline and follow-up assessments available. Because the majority of patients initiated ERT before enrollment in MARS, the numbers of patients with baseline assessments for certain outcomes were limited, including analyses of PROs; it should be noted, however, that real-world PRO data in adults with MPS IVA are very limited, and the adult MARS population represents one of very few cohorts with data of this nature. Additionally, because data collection was limited by assessments performed during routine care, certain disease characteristics and outcomes of interest were not assessed, including phenotype and cardiac function. Moreover, in assessing comparisons of these findings with those from the 6-year analysis of the overall MARS population [19], it should be noted that the patients initiating ERT as adults included in the present analysis represent a sub-population of the 6-year cohort.

## Conclusion

In this real-world analysis of elosulfase alfa in adults with MPS IVA who initiated treatment at ≥ 18 years of age, we observed a sustained reduction in uKS and a safety profile consistent with the overall MARS cohort. Endurance and respiratory function appeared stable throughout follow-up. These data generally align with the findings from the previous analysis of the overall MARS cohort [19] and post hoc analyses of adults in the clinical trials of elosulfase alfa [14]. Continued collection and analysis of real-world data in MARS will be important to confirm the long-term benefits of initiating treatment with elosulfase alfa in adulthood and will provide the opportunity to explore the impact of ERT at different ages of treatment initiation.

## Supplementary Information

Below is the link to the electronic supplementary material.

Supplementary Material 1

## Data Availability

The de-identified individual participant data that underlie the results reported in this article (including text, tables, figures, and appendices) will be made available, together with the research protocol and data dictionaries, for non-commercial, academic purposes. Additional supporting documents may be available upon request. Investigators will be able to request access to these data and supporting documents via a website (www.BioMarin.com) beginning 6 months and ending 2 years after publication. Data associated with any ongoing development program will be made available within 6 months after approval of relevant product. Requests must include a research proposal clarifying how the data will be used, including proposed analysis methodology. Research proposals will be evaluated relative to publicly available criteria available at www.BioMarin.com to determine if access will be given, contingent upon execution of a data access agreement with BioMarin Pharmaceutical Inc.

## Abbreviations

6MWT: 6-minute walk test
ADL: Activity of daily living
AE: Adverse event
CI: Confidence interval
COVID-19: Coronavirus disease 2019
EQ-5D-5L: EuroQoL-5D-5L
ERT: Enzyme replacement therapy
FEV_1_: Forced expiratory volume in 1 s
FVC: Forced vital capacity
GALNS: *N*-acetylgalactosamine-6-sulfatase
IQR: Interquartile range
MARS: Morquio A Registry Study
MorCAP: Morquio A Clinical Assessment Program
MPS: Mucopolysaccharidosis
ND: Not determined
NH: Natural history
PRO: Patient-reported outcome
QoL: Quality of life
SAE: Serious adverse event
SD: Standard deviation
uKS: Urinary keratan sulfate
