# Supplementary material for: Long-term outcomes of elosulfase alfa enzyme replacement therapy in adults with MPS IVA: a sub-analysis of the Morquio A Registry Study (MARS)
Source: Orphanet J Rare Dis. 2025 Oct 30;20:548. doi: 10.1186/s13023-025-04064-w (PMC12574230; doi:10.1186/s13023-025-04064-w)
Supplement: Supplementary file 1 — Supplementary Material 1 [file 13023_2025_4064_MOESM1_ESM.docx]

**Supplemental data**


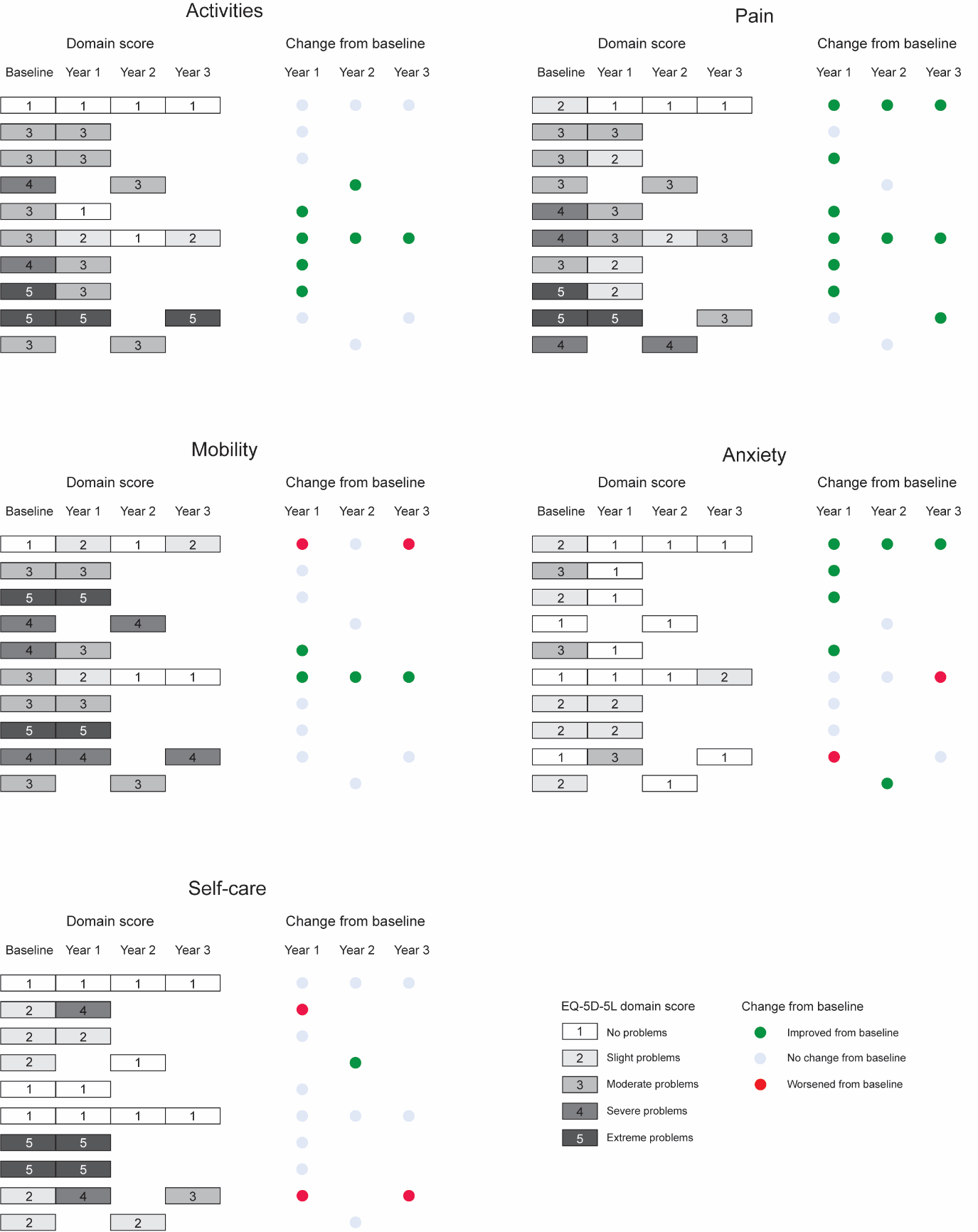


**Supplemental Fig. S1.** Characterization of change from baseline in component EQ-5D-5L domains over duration of treatment

Abbreviation: EQ-5D-5L = EuroQoL-5D-5L
